# Scale separated low viscosity dynamos and dissipation within the Earth’s core

**DOI:** 10.1038/s41598-018-30864-1

**Published:** 2018-08-22

**Authors:** Andrey Sheyko, Christopher Finlay, Jean Favre, Andrew Jackson

**Affiliations:** 1 Earth and Planetary Magnetism Group, Institute of Geophysics, ETH Zurich, Zürich, Switzerland; 2 Division of Geomagnetism, DTU Space, Technical University of Denmark, Lyngby, Denmark; 3 Swiss National Supercomputing Centre, Lugano, Switzerland

## Abstract

The mechanism by which the Earth’s magnetic field is generated is thought to be thermal convection in the metallic liquid iron core. Here we present results of a suite of self-consistent spherical shell computations with ultra-low viscosities that replicate this mechanism, but using diffusivities of momentum and magnetic field that are notably dissimilar from one another. This leads to significant scale separation between magnetic and velocity fields, the latter being dominated by small scales. We show a zeroth order balance between the azimuthally-averaged parts of the Coriolis and Lorentz forces at large scales, which occurs when the diffusivities of magnetic field and momentum differ so much, as in our model. Outside boundary layers, viscous forces have a magnitude that is about one thousandth of the Lorentz force. In this dynamo dissipation is almost exclusively Ohmic, as in the Earth, with convection inside the so-called tangent cylinder playing a crucial role; it is also in the “strong field” regime, with significantly more magnetic energy than kinetic energy (as in the Earth). We finally show a robust empirical scaling law between magnetic dissipation and magnetic energy.

## Introduction

Earth’s dynamo is generally considered to be driven by cooling of the core (radius *r* = *r*_0_) and from latent heat and buoyancy associated with the crystallisation of the inner core (radius *r* = *r*_*i*_). Complex motions **v** associated with this cooling mechanism act in concert with an existing magnetic field **B** to generate electrical currents by Faraday’s Law, and these currents generate more magnetic field by Ampere’s Law. This general picture of a magnetic field generator is termed a dynamo, but the actual details are more complex: the system is governed by the coupled momentum (Navier-Stokes), induction (pre-Maxwell) and energy equations that must be simultaneously satisfied. Of primary importance in the momentum equation is the presence of the Coriolis force, associated with the rapid rotation of the Earth; this effect is generally considered paramount in leading to a roughly dipolar field whose axis is located close to the rotation axis of the Earth.

**Figure 1:**
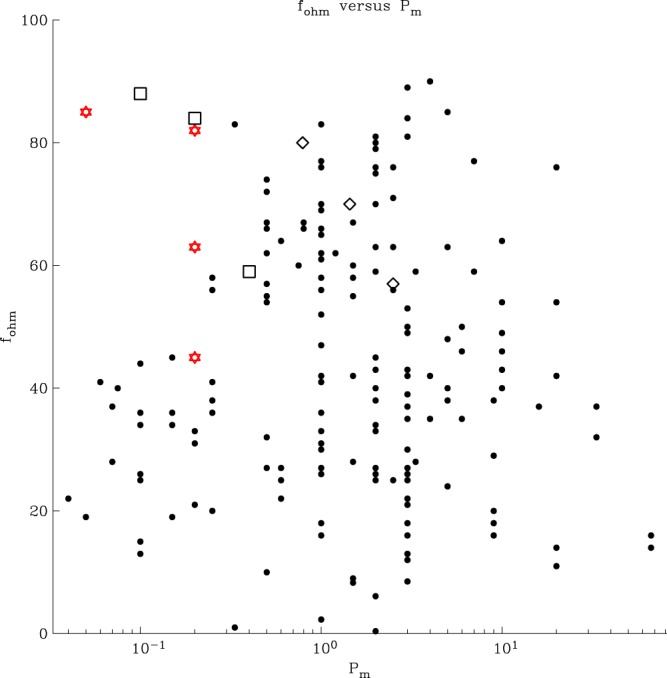
Fraction of dissipation that is Ohmic. The fraction is *f*_ohm_ = *D*_*mag*_/(*D*_*mag*_ + *D*_*kin*_) and is considered to be close to unity in planetary cores. Our models (red stars) fill the low-*Pr*_*m*_ parameter space while dissipating primarily Ohmically. Other models from the literature are shown: black dots^3,4^; open diamonds^6^ (omitting models using hyperviscosity); open squares^5^.

Numerical solutions of these sets of equations^1,2^ have borne great fruit, but numerical limitations restrict the reality of the computations performed thus far. Here we report on solutions closer to the geophysical regime, in which the Coriolis and Lorentz forces are dominant, viscosity plays a minor role, and we observe scale separation between magnetic and velocity fields. Using a length scale *L* = *r*_*o*_ − *r*_*i*_, fluid kinematic viscosity *ν* and rotation rate Ω, the Ekman number *E* = *ν*/(2Ω*L*^2^) for these simulations is as low as *E* = 3 × 10^−7^, rarely achieved in numerical studies. Reduced values of *E* imply finer flow structures, which lead to larger space and time resolutions and, as a consequence, tremendously increase computational costs. Table 1 gives details of the dynamo solutions computed, together with one purely hydrodynamic simulation (HYDRO0) that removes the presence of the magnetic field altogether (see also Supporting Figs S0–S4). A critical parameter is the magnetic Prandtl number *Pr*_*m*_ = *ν*/*η*, where *η* is the magnetic diffusivity. Liquid metals in general, including at high pressure, have very small values for *Pr*_*m*_, namely *O*(10^−5^–10^−6^). Our simulations include some that reduce this value to 0.05 in the case of model S4. The aim of the reduction in *Pr*_*m*_ is to ensure that almost all energy is dissipated Ohmically, as in the Earth, rather than viscously. Figure 1 compares our models with others in the literature^3–6^ and shows that we have achieved this, with model S4 dissipating 85% of its energy Ohmically (this fraction is commonly called *f*_ohm_).

**Table 1:** Control parameters of the numerical simulations.

Name	*E* /10^−6^	*Ra*/*Ra*_*c*_	*q* = *Pr*_*m*_	*R*_*m*_	*Re*
S0^*a*^	1.1834	14.4	0.20	63	315
HYDRO0	1.1834	14.4			457
S1	1.1834	72	0.20	180	900
S2	1.1834	432	0.20	868	4340
S4	0.2959	432	0.05	274	5480

*E* is the Ekman number, *Ra* is the Rayleigh number, *Pr*_*m*_ is the magnetic Prandtl number. Output characteristics are the magnetic Reynolds number *R*_*m*_ and the conventional Reynolds number *Re*. The Prandtl number is unity for all runs. Ra_c_ is the critical Rayleigh number for the onset of non-magnetic convection.

The Rayleigh number is measured relative to the critical Rayleigh number for the onset of convection, *Ra*_*c*_, and the Prandtl number *Pr* = 1.

^*a*^S0 is as in ref.^8^ but with ten times lower *Ra*.

Our simulations in the electrically-conducting fluid outer and solid inner cores (*r*_*i*_/*r*_*o*_ = 0.35) are constructed with the energy to drive the dynamos partitioned equally between heating at the inner core boundary (ICB; simulating crystallisation) and internal heating (secular cooling)^7^. We use constant heat flux boundary conditions^8^ at the core-mantle boundary (CMB) and a constant temperature ICB; no-slip boundary conditions are applied at the ICB and CMB and the inner core is conducting and free to rotate. Details of our computations, which are standard, are given in the Materials and Methods section. Kinetic Reynolds numbers in excess of 5000 are achieved in our most extreme calculation S4; see Table 1. The unit for magnetic field we use is the “Elsasser unit” $\sqrt{2\rho {\rm{\Omega }}{\mu }_{0}\eta }$ where *ρ* and *μ*_0_ are density and free space magnetic permeability respectively. With the recent values^9^ for *η* of approximately 0.5 *m*^2^ *s*^−1^, one Elsasser unit is close to 1 mT. We have run the simulations, which are computationally demanding (requiring more than 1700 CPU years), until a statistical equilibrium is found. All of our solutions are quasi-steady, dipole-dominated and non-reversing.

**Figure 2:**
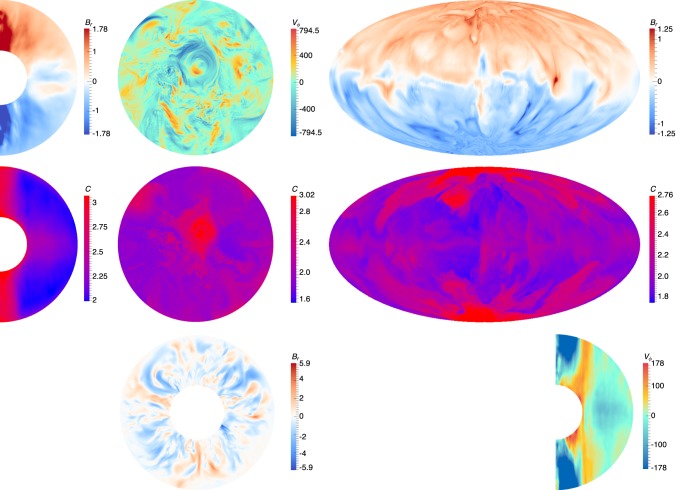
Structures of velocity, magnetic and temperature fields in Case S4. The left hand column shows meridional sections (quantities have been averaged in time and azimuth), the middle column shows views from above of the plane z = *r*_*i*_ + 0.5, and the third column shows the surface of the sphere in a Hammer projection (all these are at *t* = 0.0712). The top row shows radial magnetic field *B*_*r*_, azimuthal velocity v_*ϕ*_ and radial field *B*_*r*_ at *r*_*o*_. Second row shows temperature with red representing higher temperature. The last row shows radial magnetic field in the equatorial plane at *t* = 0.0712324 and azimuthal velocity v_*ϕ*_ averaged in azimuth and time.

Fixed heat flux boundary conditions were suggested by Sakuraba and Roberts^8^ as being more realistic boundary conditions for the core considering the overlying convecting mantle. They showed that the use of these boundary conditions affected the length scale of magnetic fields. Our simulation S0 has the same values of *E*, *Pr* and *Pr*_*m*_ as^8^, but 10 times lower Ra [This corrects an incorrect statement made in^10^ that S0 had the same *Ra* as^8^, this error of comparison was caused by inappropriate non-dimensionalizations but does not affect any of the results reported by^10^. We thank N. Schaeffer for pointing out this error]. Case S0 exhibits westward drift at the equator as a result of thermal winds driven by development of a hot equator. Convection has not set in within the tangent cylinder (the imaginary cylinder just touching the inner core and parallel to the axis of rotation). When the Rayleigh number is raised by a factor of five, Case S1 exhibits convection within the tangent cylinder and reduced CMB temperature gradients, with concomitant reduced westward drift. As the Rayleigh number is raised to thirty times that of Case S0, the sign of the temperature gradient is reversed, leading to a cold equator, hot poles and a weak eastward drift. Case S4, which represents our most extreme calculation, has the same Rayleigh number as Case S2 but has had its Ekman and magnetic Prandtl numbers reduced by factors of four. Now (see Fig. 2) the tangent cylinder is very hot, but temperature gradients outside the TC are ameliorated to an extent that there is essentially no global azimuthal flow at the equator of the CMB. Thus, aside from the thermal boundary condition, the supercriticality of the Rayleigh number plays a vital role in determining whether there is azimuthal flow at the equator or not.

**Figure 3:**
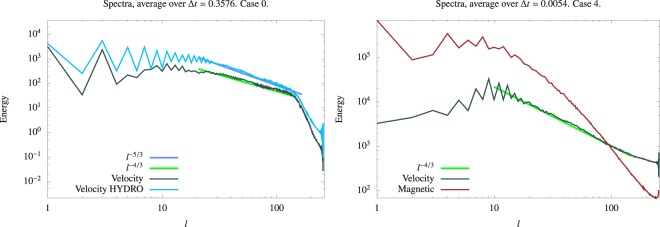
Time-averaged energy spectra. (**a**) Kinetic energy spectra (volume averaged) as a function of spherical harmonic degree *l* are compared for Case S0 (a dynamo) and Case HYDRO0: we see that the Lorentz force modifies the velocity field at all scales from *l* = 1 to *l*~150. (**b**) Magnetic and kinetic energy spectra as a function of spherical harmonic degree *l* for Case S4. The Ekman number is 3 × 10^−7^ and *Pr*_*m*_ = 0.05. There is scale separation between the large scale magnetic field peaking at *l* = 1 and the velocity field with maximum energy at *l* = 9. Note that the magnetic energy, which is ten times larger in total than the kinetic energy, exceeds the latter for all *l* < 100.

Our simulations show evidence for the role of the Lorentz force in modifying the flow at all scales. Figure 3(a) compares non-magnetic and magnetic simulations at the same Rayleigh, Ekman and Prandtl numbers (Case S0 and Case HYDRO0).

The magnetic field generates a Lorentz force that substantially modifies the energy content at all scales. Note that in the HYDRO0 case we have a very good fit to Kolmogorov’s −5/3 law for the energy spectrum, despite the fact that this law is generally applied to isotropic non-rotating turbulence. Figure 3(b) shows our extreme simulation at *E* = 3 × 10^−7^, *Pr*_*m*_ = 0.05 (Case S4). Magnetic energy exceeds kinetic energy for all spherical harmonic degrees *l* < 100. When the kinetic spectrum is modified by the presence of magnetic field, it fits a −4/3 slope in both Case S0 and Case S4.

## Force Balance

**Figure 4:**
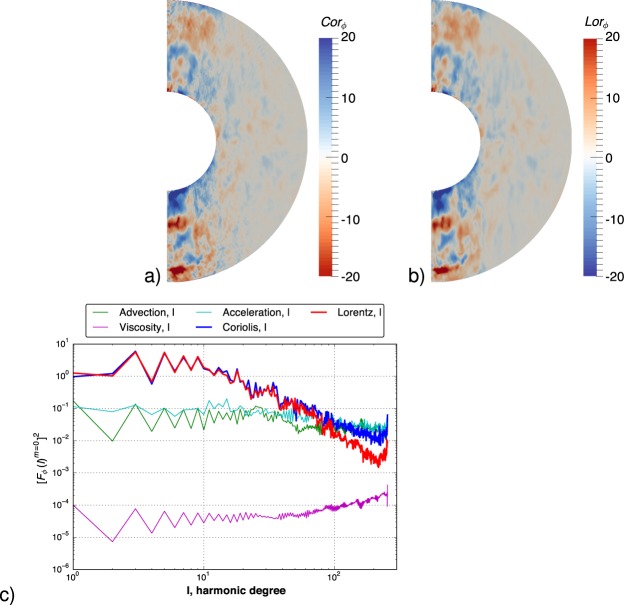
Instantaneous force balances in model S4 at *t* = 0.174. (**a**,**b**) show the azimuthally averaged *ϕ* component of the Coriolis and Lorentz forces respectively, where the Coriolis force has an inverted color bar to aid comparison. For this average the pressure gradient vanishes identically and thus one can see almost perfect balance between the forces. (**c**) Shows the same azimuthal component of all forces occurring in the Navier-Stokes equation when averaged over azimuth, squared, and integrated in non-dimensional radius (omitting 10% in radius at each boundary) as a function of spherical harmonic degree. This again removes the pressure component. The choice of radii is such that it excludes the boundary layers where there is a viscous-Coriolis-Lorentz balance. At the largest scales the balance between Coriolis and Lorentz forces is almost perfect. The rms viscous force is about 3 orders of magnitude smaller at the largest scales.

The Coriolis and Lorentz forces in our most extreme model, Case S4, are shown in Fig. 4(a–c). Since convection is well-developed within the tangent cylinder, the forces are strongest here. In the figures we choose to examine the azimuthal average of the azimuthal component of all forces, which immediately removes the contribution from the pressure and buoyancy. We find evidence of a primary local balance between Coriolis and Lorentz forces, essentially the magnetostrophic or MAC force balance foreseen by Taylor^11^, and reported in some other very recent low *E* simulations^5,6,12,13^. The use of a particularly small Ekman number means that the viscous forces play an insignificant role in the bulk of our dynamo, as expected for the Earth’s core. While we see the largest scales of the azimuthal average of the instantaneous Coriolis and Lorentz forces are almost identical in Fig. 4a,b, there is a slight mismatch at the very smallest scales. To see this more clearly we examine the same quantity in the spectral domain, and examine the contributions by spherical harmonic degree^6^. Figure 4c shows a remarkable zeroth order balance between the azimuthally-averaged parts of the Coriolis and Lorentz forces at large scales, but at small scales that balance has been broken. This effect arises when the diffusivities of magnetic field and momentum differ so much, as in our model. The Coriolis force is small scale, as it depends on **u**, whereas the Lorentz force is larger scale, depending quadratically on **B**. Although large scales can be balanced, the scale separation leads to a different balance at small scales where the role of nonlinear advection (Reynolds stresses) and accelerations are more pronounced. Even at the smallest scales the viscous force remains one order of magnitude smaller (in rms) than the Coriolis force. We believe that this behaviour represents well the appropriate dynamics of the core. Yadav *et al*.^12^ have also recently presented evidence of a time-averaged Lorentz-Buoyancy-Coriolis balance at an Ekman number of 5 × 10^−7^ (using our definition of the Ekman number), albeit with a 2 > *Pr*_*m*_ > 0.4, so that their scale separation is not as prominent.

## Dissipation

All of our simulations have magnetic energy exceeding kinetic energy as is expected in the Earth’s core, see Table 2. Of particular interest is the way that energy is dissipated. A fundamental study of this was carried out by^14^. Their study noted the inverse scaling of dissipation with magnetic Reynolds number, and used an estimate of the latter for the Earth to deduce a value of 0.5 TW for the dissipation taking place in the Earth’s core. Here we take a different approach that focuses on the internal magnetic energy rather than the magnetic Reynolds number.

**Table 2:** Diagnostics measured in the simulations.

	*E*_*mag*_	*E*_*kin*_	*E*_*mag*_/*E*_*kin*_	*D*_*mag*_	*D*_*kin*_	*D*_*mag*_/*D*_*kin*_
S0	8.71e + 04	2.91e + 04	2.99	3.41e + 07	4.04e + 07	0.84
S1	7.50e + 05	2.38e + 05	3.15	8.66e + 08	5.04e + 08	1.72
S2	9.52e + 06	5.50e + 06	1.73	4.85e + 10	1.18e + 10	4.11
S4	2.13e + 06	5.50e + 05	3.87	3.34e + 09	5.73e + 08	5.83

*E*_*mag*_ and *E*_*kin*_ are the magnetic and kinetic energies, while *D*_*mag*_ and *D*_*kin*_ are the Ohmic and viscous dissipations.

**Figure 5:**
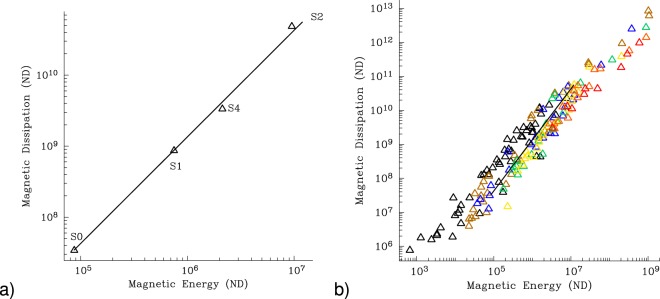
Variation of magnetic dissipation *D*_*mag*_ with magnetic energy *E*_*mag*_. (**a**) for the new simulations reported herein (see Table 1) and (**b**) for the dataset from Uli Christensen^3,4^. In (a) the solid line is a law of the form ${D}_{mag}=1.35{E}_{mag}^{3/2}$, and fits all three *Pr*_*m*_ = 0.2 models. In (b) colours indicate the fraction of energy dissipated Ohmically. The brightening spectrum starts with models (black) with predominantly viscous dissipation (Ohmic dissipation ≤30%) and proceeds black-brown-blue-green-yellow-orange-red to the models dissipating mostly (red: 80–90%) Ohmically. The straight line is a law of the form ${D}_{mag}=1.35{E}_{mag}^{3/2}$.

Ohmic dissipation dominates our simulations as the mechanism by which energy is returned to heat, with the exception of Case S0 where viscous and Ohmic dissipations are close to being equal. In Case S4 the effect of the well-developed convection in the tangent cylinder leads to a remarkable concentration of the Ohmic dissipation in this area (see Supporting Fig. S1). Analysis of the distribution of dissipation shows that it is relatively constant in radius. When we analyse all our models together we observe a very precise power law scaling of magnetic dissipation with magnetic energy that appears to remain true over 3 decades of magnetic energies (Fig. 5a).

The favoured scaling fit to these non-dimensional data is of the form ${D}_{mag}=1.35{E}_{mag}^{3/2}$. We compare this law to the large library of runs supplied by Uli Christensen^3,4^ in Fig. 5b. [We have rescaled all the results so that time is measured in terms of Ohmic decay times. This leads to Christensen’s results being scaled by $V\,{P}{{r}}_{m}^{a}$, where *V* is the shell volume and *a* = 3 for dissipation and *a* = 2 for energy]. One can see that there is reasonable consistency of the fit to the scaling law. The power law can be argued to be consistent with a previous theory^3,15^ (see Supporting Information).

## Materials and Methods

### Governing equations and non-dimensionalization

We adopt the Boussinesq approximation for convection-driven, rotating magnetohydrodynamics, which results in the following non-dimensional equations:

1 $$\begin{array}{ccc}(Ro\,\frac{{\rm{\partial }}}{{\rm{\partial }}t}-E{{\boldsymbol{\nabla }}}^{2})\,{\bf{v}} & = & {{\bf{N}}}_{{\rm{v}}}-\,{\boldsymbol{\nabla }}\,\hat{P},\\ (\frac{{\rm{\partial }}}{{\rm{\partial }}t}-{{\boldsymbol{\nabla }}}^{2})\,{\boldsymbol{B}} & = & {\boldsymbol{\nabla }}\times ({\bf{v}}\times {\boldsymbol{B}}),\\ (\frac{{\rm{\partial }}}{{\rm{\partial }}t}-q\,{{\boldsymbol{\nabla }}}^{2})\,T & = & \varepsilon -{\bf{v}}\cdot {\boldsymbol{\nabla }}\,T,\end{array}$$

where

$${{\bf{N}}}_{{\rm{v}}}=Ro\,{\bf{v}}\times ({\boldsymbol{\nabla }}\times {\bf{v}})+({\boldsymbol{\nabla }}\times {\bf{B}})\times {\bf{B}}+q\,Ra\,T\,{\bf{r}}-\hat{{\bf{z}}}\times {\bf{v}}.$$

Variables **v**, **B**, *T* are the velocity, magnetic field and temperature. ${\boldsymbol{\nabla }}\hat{P}$ is the modified pressure that contains information about conservative forces. The axis of rotation of the system is *z* and $\hat{{\bf{z}}}$ is a unit vector in its direction. Time is denoted as *t*. A uniform heat source *ε* is included. Incompressibility conditions ${\boldsymbol{\nabla }}\cdot {\bf{B}}=0$ and ${\boldsymbol{\nabla }}\cdot {\bf{v}}=0$ are integrated into the solution technique through use of a poloidal-toroidal decomposition of the vector field. Non-dimensional parameters are defined as:

2 $$\begin{array}{ll}{\rm{Magnetic}}\,{\rm{Rossby}}\,{\rm{number}} & Ro=\eta /(2{\rm{\Omega }}{L}^{2}),\\ {\rm{Ekman}}\,{\rm{number}} & E=\nu /(2{\rm{\Omega }}{L}^{2}),\\ {\rm{Modified}}\,{\rm{Rayleigh}}\,{\rm{number}} & Ra=g\,\alpha \,{\rm{\Delta }}T\,L/(2{\rm{\Omega }}\kappa ),\\ {\rm{Roberts}}\,{\rm{number}} & q=\kappa /\eta .\end{array}$$

The units of length, time, magnetic field and temperature for the non-dimensional governing equations are chosen as follows:

$$r\to L\,r,\,t\to {L}^{2}/\eta \,t,\,B\to {(2{\rm{\Omega }}\rho {\mu }_{0}\eta )}^{\frac{1}{2}}\,B,\,$$

$$T\to {\rm{\Delta }}T\,T,\,L={r}_{o}-{r}_{i}\,.$$

The following symbols denote the parameters of the system: ${\boldsymbol{\Omega }}={\rm{\Omega }}\hat{{\bf{z}}}$ is the rotation rate, *μ*_0_ is the permeability of free space, *ρ* is the density, Δ*T* is the unit of temperature, *ν*, *κ* and *η* are the kinematic viscosity, thermal diffusivity and magnetic diffusivity respectively, and *α* is the thermal expansivity. Gravity is assumed to vary linearly with radius and has value g on the outer boundary. The spherical coordinates are denoted (*r*, *θ*, *ϕ*).

### Boundary conditions and internal heating

The modelled fluid is enclosed in a rotating spherical shell between radii *r*_*i*_ and *r*_*o*_ with *c* = *r*_*i*_/*r*_*o*_ = 0.35. Both boundaries are no-slip and impermeable. The outer boundary is electrically insulating, the inner core has the same electrical conductivity as the outer core. The inner core temperature is kept constant at *T* = 5.434, the gradient of temperature on the outer core equals to −2/(1 − *c*). A uniform heat source with *ε* = 3*q* is adopted throughout the outer core. For details see^10^.

### Diagnostics

The kinetic and magnetic energies are defined as ${E}_{kin}=\frac{1}{2}\,\int \,{{\rm{v}}}^{2}{\rm{d}}{\rm{V}}$ and ${E}_{mag}=\frac{1}{2Ro}\,\int \,{B}^{2}{\rm{dV}}$, where the volume integral is over the entire outer core. The viscous and Ohmic dissipations are defined as ${D}_{kin}=\frac{E}{Ro}\,\int \,{({\rm{\nabla }}\wedge {\bf{v}})}^{2}{\rm{d}}{\rm{V}}$ and ${D}_{mag}=\frac{1}{Ro}\,\int \,{(\nabla \wedge {\bf{B}})}^{2}{\rm{dV}}$. The magnetic Reynolds number *R*_*m*_ is vL/*η*.

### Numerical setup

We solve the governing equations using a parametrization in spherical harmonics up to degree and order 255 for the angular component and 528 finite difference points in radius. A second order predictor-corrector scheme is used for the time integration^16^. The timestep is adaptive and varies throughout the run. Parallelisation is carried out in radius. In the linear parts of the code, data is split over the spherical harmonics. 528 cores were used simultaneously for one simulation. The bulk of the simulations and visualizations were performed on the supercomputer s Piz Daint (Cray XC 30) and Monte Rosa (Cray XE 6) at Swiss National Supercomputing Center. The code was originally developed by Willis^17^ and then subsequently optimized for the Cray and successfully benchmarked against other dynamo codes^18^.

## Electronic supplementary material

Supplementary Information

## Data Availability

Data used for plots can be supplied by the authors upon request.
