# Supplementary material for: Scale separated low viscosity dynamos and dissipation within the Earth’s core
Source: Sci Rep. 2018 Aug 22;8:12566. doi: 10.1038/s41598-018-30864-1 (PMC6105638; doi:10.1038/s41598-018-30864-1)
Supplement: Supplementary file 1 — Supplementary Information [file 41598_2018_30864_MOESM1_ESM.pdf]

Scale separated low viscosity dynamos and dissipation within the Earth's core

Andrey Sheyko[1], Christopher Finlay[2], Jean Favre[3] and Andrew Jackson[1]*

1. Earth and Planetary Magnetism Group, Institute of Geophysics, ETH Zurich.

2. Division of Geomagnetism, DTU Space, Technical University of Denmark.

3. Swiss National Supercomputing Centre, Lugano, Switzerland.

* Corresponding Author

Supplementary Information

The dissipation-energy relation Here we remark that the scaling relationship $D_{mag} \sim 1.35 E_{mag}^{3/2}$ can be related to the magnetic field scaling laws [2,3]. The Ohmic dissipation is related to the energy flux Fq_0 by [2]

$$D = f_{\rm ohm} F q_0. \tag{S 1}$$

The Lorentz number scaling of **B** with Fq_0 is very close to $1/3$ and so magnetic energy will scale with a $2/3$ exponent. Using (S 1) with $f_{\rm ohm} = 1$ gives $E_{mag} \sim D_{mag}^{2/3}$, leading to the required relationship. We note that stress-free calculations [4] give a B-scaling exponent of 0.37. One can

see that a relationship $D_{mag} \sim E_{mag}^{4/3}$ would lead to a scaling exponent of $3/8 = 0.375$ which is essentially the same. However this law is not really preferred by our data, instead we find more accord with the original exponent [3]. Thirty-eight out of forty of the models presented in Yadav et al. [4] have $D_{kin} > D_{mag}$ and thus do not dissipate primarily through Ohmic dissipation. This may contribute to the discrepancy.

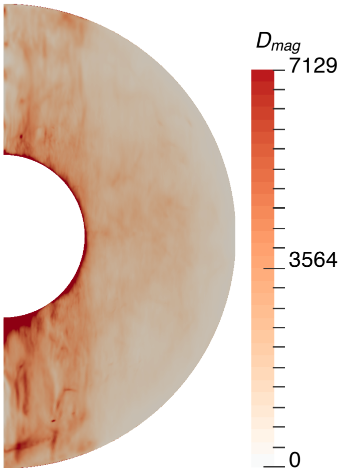

Figure S 1: **Azimuthally averaged Ohmic dissipation for model S4.** The importance of the tangent cylinder is apparent. Dissipation within the inner core is not plotted. On account of the small volume of the inner core, this amounts to only a few percent of the total dissipation.

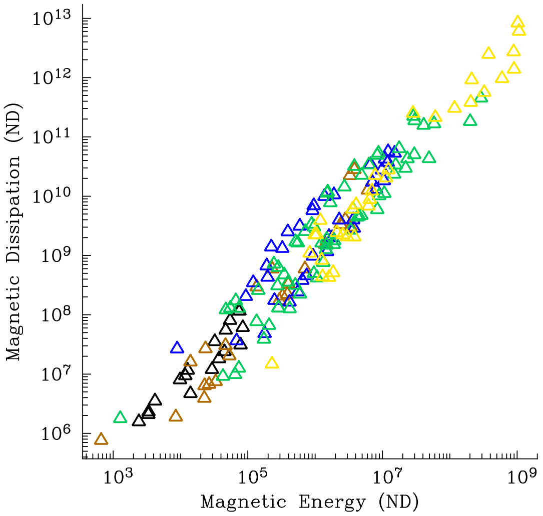

Figure S 2: **Systematics in Ohmic dissipation from the database of U. Christensen [1,3]**. Magnetic energy versus dissipation. Colours indicate the value of Pr_m: black < 0.15; brown 0.2-0.5; blue 0.6-1; green 1.2-4; yellow 5-66. One can see clearly the trend of dissipations *increasing* as Pr_m decreases.

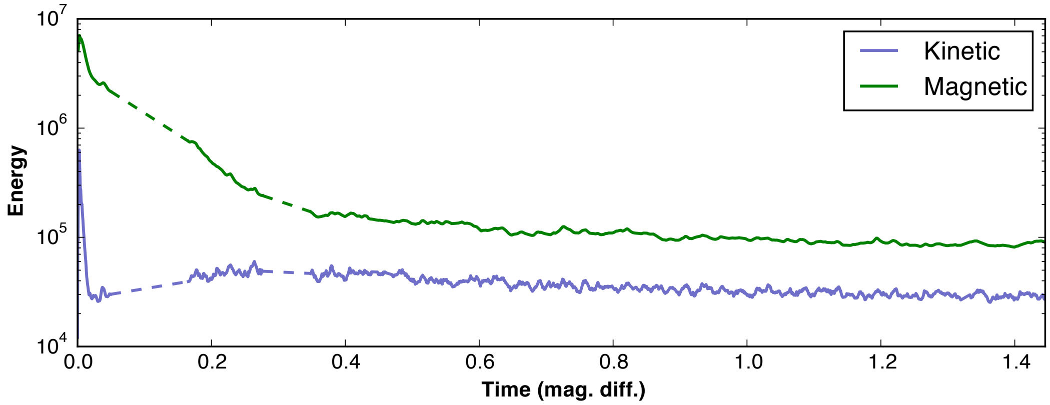

Figure S 3: **Magnetic and kinetic energy dependencies for dynamo S0. Dashed lines show interpolations where data are not recorded. Averages are taken over the period** $1.3325766 \leq t \leq 1.4454470$**.**

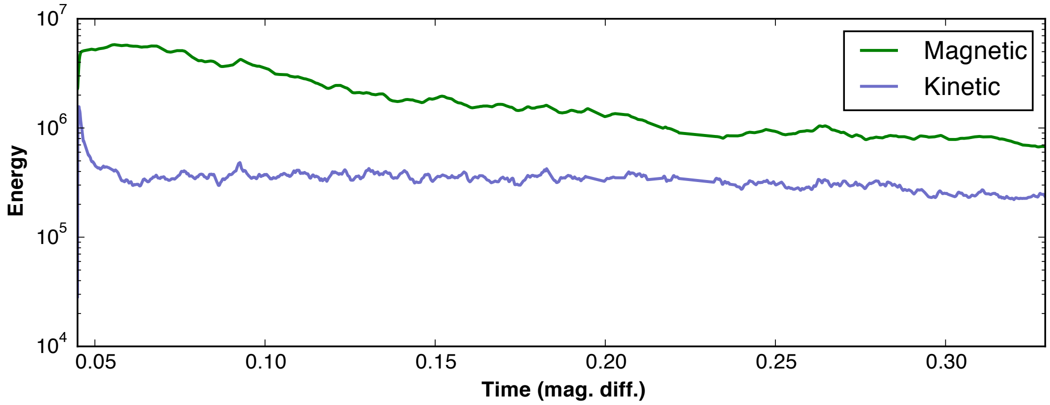

Figure S 4: **Magnetic and kinetic energy dependencies for dynamo S1. Averages are taken over the period** $0.3104368 \leq t \leq 0.3292763$**.**

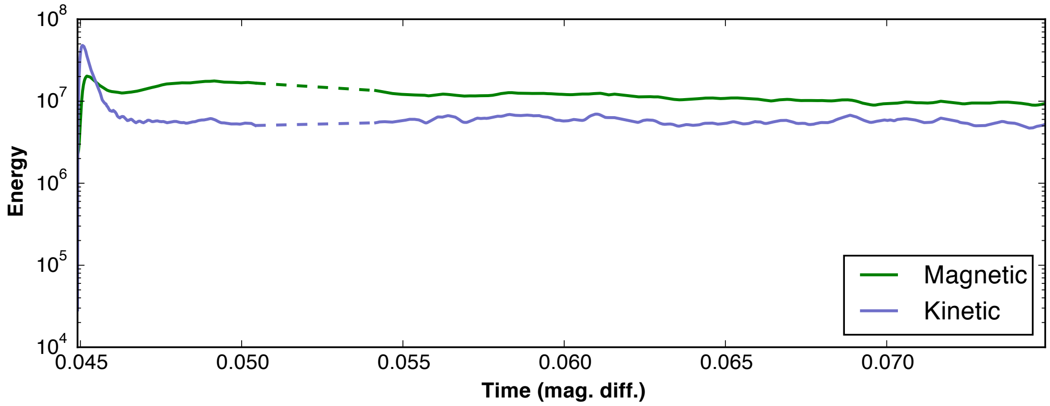

Figure S 5: **Magnetic and kinetic energy dependencies for dynamo S2. Dashed lines show interpolations where data are not recorded. Averages are taken over the period** $0.0700006 \leq t \leq 0.0749146$**.**

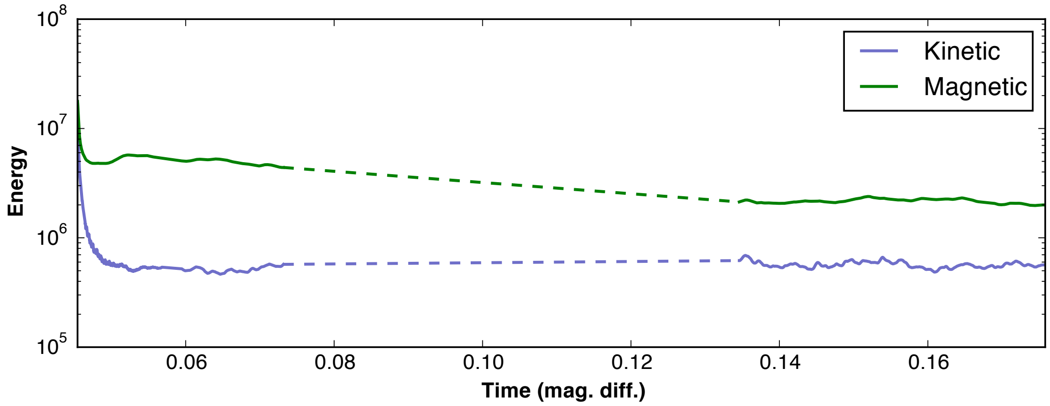

Figure S 6: **Magnetic and kinetic energy dependencies for dynamo S4. Dashed lines show interpolations where data are not recorded. Averages are taken over the period** $0.1600050 \leq t \leq 0.1758150$**.**

1. Ulrich R Christensen, Volkmar Holzwarth, and Ansgar Reiners. Energy flux determines magnetic field strength of planets and stars. *Nature*, 457(7226):167–169, 2009.

2. UR Christensen. Dynamo scaling laws and applications to the planets. *Space Science Reviews*, 152(1-4):565–590, 2010.

3. UR Christensen and Julien Aubert. Scaling properties of convection-driven dynamos in rotating spherical shells and application to planetary magnetic fields. *Geophysical Journal International*, 166(1):97–114, 2006.

4. Rakesh K Yadav, Thomas Gastine, and Ulrich R Christensen. Scaling laws in spherical shell dynamos with free-slip boundaries. *Icarus*, 225(1):185–193, 2013.
